# The first complete genome sequences of the acI lineage, the most abundant freshwater *Actinobacteria*, obtained by whole-genome-amplification of dilution-to-extinction cultures

**DOI:** 10.1038/srep42252

**Published:** 2017-02-10

**Authors:** Ilnam Kang, Suhyun Kim, Md. Rashedul Islam, Jang-Cheon Cho

**Affiliations:** 1 Department of Biological Sciences, Inha University, Incheon 22212, Republic of Korea

## Abstract

The acI lineage of the phylum *Actinobacteria* is the most abundant bacterial group in most freshwater lakes. However, due to difficulties in laboratory cultivation, only two mixed cultures and some incomplete single-amplified or metagenome-derived genomes have been reported for the lineage. Here, we report the initial cultivation and complete genome sequences of four novel strains of the acI lineage from the tribes acI-A1, -A4, -A7, and -C1. The acI strains, initially isolated by dilution-to-extinction culturing, eventually failed to be maintained as axenic cultures. However, the first complete genomes of the acI lineage were successfully obtained from these initial cultures through whole genome amplification applied to more than hundreds of cultured acI cells. The genome sequences exhibited features of genome streamlining and showed that the strains are aerobic chemoheterotrophs sharing central metabolic pathways, with some differences among tribes that may underlie niche diversification within the acI lineage. Actinorhodopsin was found in all strains, but retinal biosynthesis was complete in only A1 and A4 tribes.

Considering the influence of inland waters on global climate change^1^,^2^ and the essential roles of microbes in biogeochemical processes^3^, studies on major bacterial groups in freshwater environments are important. The acI lineage of the phylum *Actinobacteria*, comprised of ~13 tribes belonging to acI-A, -B, or -C sublineages, represents one of the most widespread and abundant bacterial groups in freshwater environments^4^,^5^. Since the arbitrary naming based on phylogenetic analyses of 16 S rRNA gene clone sequences retrieved from freshwater environments^6^, the acI clade has been widely found in diverse freshwater habitats^7^,^8^,^9^ by using various approaches^10^,^11^,^12^. Through the taxonomic binning of metagenome sequences from lakes and estuaries, it was found that genomes of several freshwater actinobacterial lineages including the acI and acIV clades have low GC contents^13^. Studies based on fluorescence *in situ* hybridization combined with microautoradiography have shown that the acI members can uptake thymidine, glucose, leucine, and amino acid mixture^11^,^14^,^15^.

However, studies on the acI lineage have been lagging, largely due to difficulties in the cultivation of acI members. Only two acI strains, affiliated with the acI-A2 and acI-B2 tribes, have been cultivated under laboratory condition as mixed enrichment cultures^16^,^17^,^18^. ‘*Candidatus* Planktophila limnetica’, was proposed to represent strain MWH-EgelM2-3, the first mixed culture of the acI clade^16^. This strain was affiliated with the acI-A2 tribe, but was never cultured in pure. The proportion of this strain in mixed cultures did not exceed 5.6%, hampering further characterization. The second isolate of the acI clade was found in an enrichment culture that was established using a medium based on sterile-filtered lake water. This acI strain, belonging to the acI-B2 tribe, occupied up to 78% of the enrichment cultures^17^. A subsequent study that applied shotgun metagenome sequencing to this enrichment cultures showed that the acI-B2 strain coexisted and interacted with at least 3 other bacterial strains^18^.

Recently, genome sequences of some acI members have been obtained by metagenome assembly followed by contig binning^19^,^20^,^21^ and sequencing of single-amplified genomes (SAGs)^22^,^23^, providing insights into ecological niches of the acI lineage. Starting with the acI-B1 tribe^22^, many SAGs of the acI clade have been reported, encompassing at least 3 tribes^23^. These acI SAGs confirmed the low GC content of abundant freshwater actinobacterial populations and also showed the acI genomes are small and streamlined. Actinorhodopsin genes were found in many acI SAGs, confirming a previous study that reported the wide distribution of actinorhodopsin gene among freshwater actinobacteria^24^. Deep metagenome sequencing also enabled the reconstruction of the acI genomes from a freshwater reservoir^19^ and brackish environments^20^,^21^. Metagenome-assembled genomes (MAGs) of the acI clade also showed the genomic features such as low GC content and small size. However, these acI genomes obtained by single cell genomics and metagenomics were incomplete due to inherent limitations of the methods employed, and could not either provide comprehensive overviews on the metabolic potentials of the acI lineage, or show differences among the tribes of the acI lineage clearly.

In the present study, we presented the first reported complete genome sequences of the acI lineage. Four strains of the acI lineages, each representing different tribes, were isolated by dilution-to-extinction culturing, and their complete genomes were obtained successfully through whole genome amplification. The acI genome sequences showed features of genome streamlining in common, and exhibited some differences among the tribes regarding metabolic potentials, which may affect the overall prevalence of the lineage and the co-occurrence of diverse tribes in freshwater habitats.

## Results

### Isolation of freshwater acI strains using dilution-to-extinction culturing

**Figure 1:**
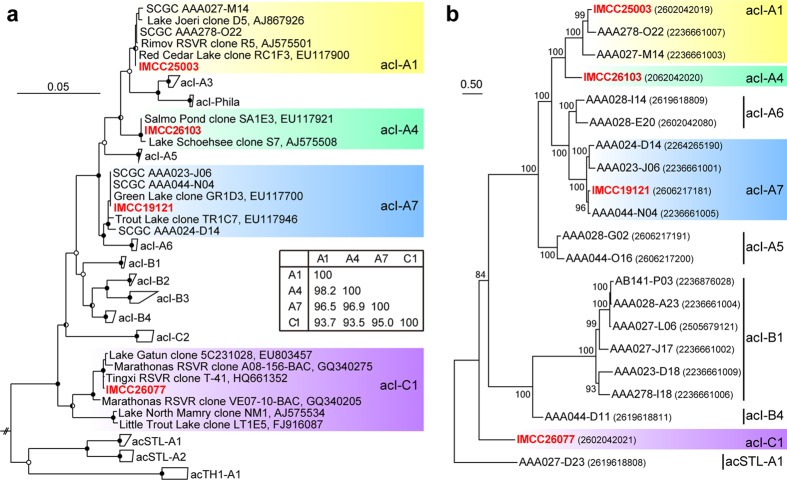
Phylogenetic analyses of the acI strains. (**a**) A maximum-likelihood tree based on 16 S rRNA gene sequences showing the phylogenetic position of the acI strains obtained in the present study. Bootstrap supporting values (from 1,000 replicates) are shown at the nodes as filled circles (≥90%), half-filled circles (≥70%), and empty circles (≥50%). *Rhodococcus opacus* (X80630) and *Streptomyces griseus* (AY999909) were used as outgroups. RSVR in clone names means reservoir. Bar, 0.05 substitutions per nucleotide position. The inset table shows the 16 S rRNA gene sequence similarities among the acI strains obtained in the present study. (**b**) A phylogenomic tree constructed using PhyloPhlAn based on conserved protein sequences. SH-like local support values are indicated at the nodes. The numbers in parentheses are genome IDs in the IMG database. The SAG AAA027-D23 was set as a root after tree construction.

During a study on the bacterioplankton community of Lake Soyang (South Korea) by the application of dilution-to-extinction high throughput culturing (HTC)^25^, several strains of the freshwater acI actinobacterial lineage were sporadically isolated, with cell densities of around 10^5^–10^6^ cells ml^−1^. Phylogenetic analysis of 16 S rRNA gene sequences amplified from the original HTC cultures showed that these acI strains belonged to diverse tribes of acI: acI-A1 (IMCC25003), acI-A4 (IMCC26103), acI-A7 (IMCC19121), and acI-C1 (IMCC26077) (Table 1 and Fig. 1a). All strains represented the first cultured isolates in each of the tribes they are affiliated with. In particular, even considering the enrichment cultures, IMCC26077 is the first isolate of the acI-C sublineage. IMCC25003 showed >99.5% 16 S rRNA gene sequence similarity to 2 acI-A1 SAGs (SCGC AAA278-O22 and SCGC AAA027-M14), and IMCC19121 was identical to 2 acI-A7 SAGs (SCGC AAA023-J06 and SCGC AAA044-N04) (Fig. 1a). Among the strains isolated in the present study, the similarity between IMCC25003 and IMCC26103 was the highest (98.2%), while IMCC19121 showed slightly lower similarities to IMCC25003 (96.5%) and IMCC26103 (96.9%). The similarity of IMCC26077 to the acI-A strains were only 93.7–95.0% (Fig. 1a; inset).

Attempts were made to establish stable cultures of acI strains under laboratory conditions without success. Strain IMCC25003 was grown successfully during revival from the glycerol stock and the first transfer, with cell densities reaching 5 × 10^5^ cells ml^−1^ after 15–20 days of incubation. However, with the next transfer, no meaningful growth was observed, even after 60 days (Supplementary Fig. S1a). IMCC26103 and IMCC19121 were outgrown by other bacterial strains during the revival, which was revealed by 16 S rRNA gene amplicon sequences (IMCC26103; Supplementary Fig. S1b) and cell morphology (IMCC19121; Supplementary Fig. S1d). Strain IMCC26077 grew to a cell density of 3 × 10^6^ cells ml^−1^ after 3 months at the first transfer, but was outgrown by a flavobacterial strain during the 2^nd^ or 3^rd^ transfer (Supplementary Fig. S1c). Together, these unsuccessful attempts to obtain stable pure cultures of acI strains, as like in previous studies^16^,^17^, implied that presently unknown factors would be required for the sustainable cultivation of the acI lineage under laboratory conditions.

### Whole genome amplification (WGA), genome sequencing, and assembly

WGA was performed for the acI strains retrieved by HTC to obtain sufficient amounts of genomic DNA for whole genome sequencing. Four microliters of glycerol stocks or frozen cultures were used for WGA by multiple displacement amplification (MDA). The number of cells used for MDA reactions ranged from ~500 (IMCC26103) to ~12,000 (IMCC26077). All reactions yielded >15 μg of amplified DNA. Restriction fragment length polymorphism (RFLP) and sequencing analysis of 16 S rRNA genes amplified from the MDA products showed that the genomes of the original acI strains were successfully amplified without significant contamination. RFLP banding patterns were same to those of the original cultures and sequencing electropherograms showed clear peaks with a very low proportion of noisy peaks.

Whole genome sequencing by the Illumina platform and assembly by SPAdes produced a single long contig for each of the four acI strains. Lengths of the single long contigs were approximately 1.35–1.55 Mbp and comparable to the genome sizes estimated from acI SAGs and MAGs^19^,^23^. Other contigs obtained from each strain were much shorter or had much lower coverage than the single long contigs, suggesting that the single long contigs represent nearly complete genomes for all strains (Supplementary Table S1). Subsequent PCR and sequencing that targeted the end regions of the contigs closed the contigs successfully, resulting in the complete circular genomes of four freshwater acI strains (Supplementary Fig. S2).

Coverages (sequencing depths) over the whole length of the complete genomes, as analyzed by mapping of sequencing reads onto the complete genomes, revealed that the use of a large number of cells in MDA resulted in a substantial decrease of amplification bias inherent to MDA (Supplementary Fig. S3), as demonstrated by other studies^26^,^27^, presumably leading to a successful assembly of the complete genomes. The average coverage over the whole genomes were between 331 and 682. Minimum and maximum coverage (per base resolution) ranged between 30–92 and 2,437–4,145, respectively (Table 1). The ratio of minimum to average coverage was around 0.1, and the ratio of maximum to average coverage was <8 for all genomes. The proportion of sequencing reads mapped to the complete genomes were >95%, except for IMCC26103 (90.8%), confirming the contamination of MDA products was negligible or at least not significant.

### Annotation and comparative analysis of genomes

The sizes of the four circularly completed genomes were 1.35–1.55 Mbp in length (Table 1). IMCC25003 (A1) had the smallest genome and IMCC26077 (C1) had the largest. The lengths of the genomes generally concurred with the estimation reported from SAGs and MAGs^19^,^23^ and among the smallest of the phylum *Actinobacteria*. GC contents (in mol%) were also relatively lower than most actinobacteria, ranging from 45.5 (IMCC19121; A7) to 51.3% (C1) (Table 1), confirming previous findings about low GC contents of the acI clade^13^,^19^,^20^,^21^,^23^.

**Figure 2:**
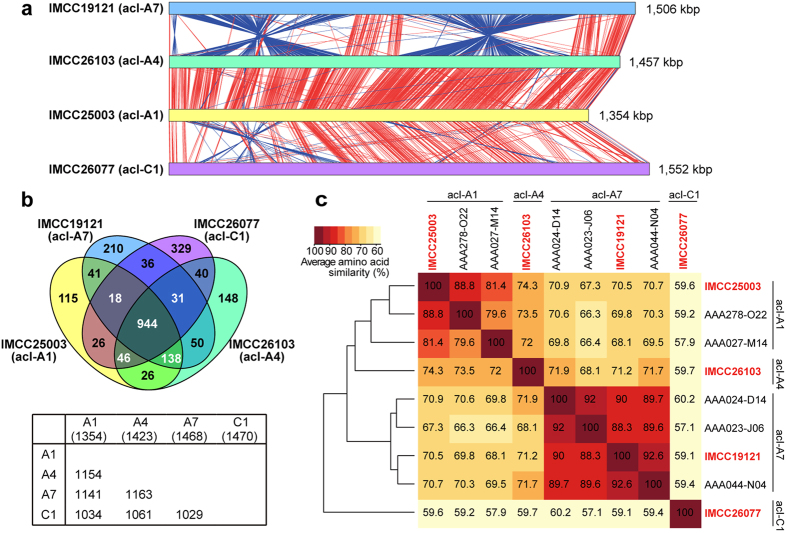
Genomic comparison of the acI genomes. (**a**) BLASTn based comparison of the acI genomes. Matches satisfying the length (≥60 bp) and score (≥50) cutoffs were displayed as red (same orientation) and blue (reverse orientation) blocks using the Artemis Comparison Tool. (**b**) Venn diagram showing the number of shared and unique protein clusters (PCs) among the acI genomes, as analyzed using GET_HOMOLOGUES. The lower table shows the number of shared PCs between the genome pairs. Note that the total number of PCs of each genome is slightly smaller than the total number of CDSs, due to putative paralogous proteins. (**c**) AAI among the acI genomes obtained in the present study and related SAGs. AAI values were obtained from GET_HOMOLOGUES. Clustering of genomes based on AAI were performed using the ‘hclust’ function of R.

Annotation through the IMG-ER pipeline showed that all the acI genomes had a single rRNA operon and 38–48 tRNA genes. The number of protein-coding genes was 1,358–1,572 (Table 1). No CRISPR locus was found in any of the genomes. A phylogenetic tree of the four complete acI genomes and some acI SAGs, constructed based on conserved proteins, showed the acI tribes defined by 16 S rRNA gene sequences remained robust in a whole-genome based analysis and corroborated the phylogenetic position of the four acI strains obtained in this study (Fig. 1b). A whole genome comparison based on BLASTn suggested that the four acI genomes shared much of their gene contents and gene order. The A1 and A4 (IMCC26103) genomes were highly similar and syntenic over the whole genome length (Fig. 2a). Compared to the A1 and A4 genomes, the A7 genome showed the possibility of two large-scale inversions but retained a high level of similarity. The genome-scale similarity of the C1 genome to the acI-A genomes was lower than similarities among the acI-A genomes (Fig. 2a).

To confirm the completeness of the acI genomes obtained in this study based on the annotation results, we utilized the 83 COG families that were used to estimate the completeness of metagenome-assembled freshwater actinobacterial genome bins^19^. All the four acI genomes were found to have a full set of 83 COG families. We also searched for the 158 conserved single-copy genes (CSCG) that were used for completeness estimation of acI SAGs^23^. The acI genomes obtained in the present study lacked only 3–5 genes. Considering that the 158 CSCGs were not completely universal (found in ≥95% of 151 complete actinobacterial genomes), this result suggested that the acI genomes of this study have little chance of being incomplete.

When the proteins encoded by the acI genomes were grouped into orthologous protein clusters (PCs) based on sequence similarity, the proportions of shared PCs between genomes were correlated with phylogenetic similarity based on 16 S rRNA gene sequences and conserved proteins (Figs 1 and 2b). The sharedness of PCs was the highest between the A1 and A4 genomes (~83%), whereas the A7 genome showed a slightly lower sharedness of 80–81% with the A1 and A4 genomes. The sharedness between the C1 genome and acI-A genomes was 70–74% (Fig. 2b). The number of PCs shared among the all acI genomes was 944, and another 138 PCs were shared among the acI-A genomes but not found in the C1 genome. The number of unique PCs increased according to genome size, from 115 (A1) to 329 (C1) (Fig. 2b). The average amino acid identity (AAI) values were 70.5–74.3% among the three acI-A genomes and 59–60% between the C1 genome and acI-A genomes, and were also proportional to sequence similarity of 16 S rRNA genes, resulting in a hierarchical clustering pattern that reflected the phylogenetic grouping of the acI tribes (Figs 1 and 2c). In terms of the correlations between 16 S rRNA gene similarity and genomic relatedness (such as proportion of shared genes and AAI values), the acI genomes showed rather lower AAI values and rather higher proportion of shared genes for their 16 S rRNA gene sequence similarities, when compared to a general pattern observed from a large number of bacterial genomes^28^,^29^. With respect to this feature, the acI strains were similar to the genus *Prochlorococcus* and the SAR11 clade, two marine bacterial groups known for their small and streamlined genomes^30^. *Prochlorococcus marinus* strains with >97% of 16 S rRNA gene similarities showed rather lower AAI values of ~60% (ref. 28). SAR11 strains of diverse subclades (~18% divergence in 16 S rRNA gene sequences) shared more than 50% of their genes, and SAR11 subclade Ia strains with >98% of 16 S rRNA gene similarities exhibited the AAI values of slightly higher than 70% (ref. 31).

**Figure 3:**
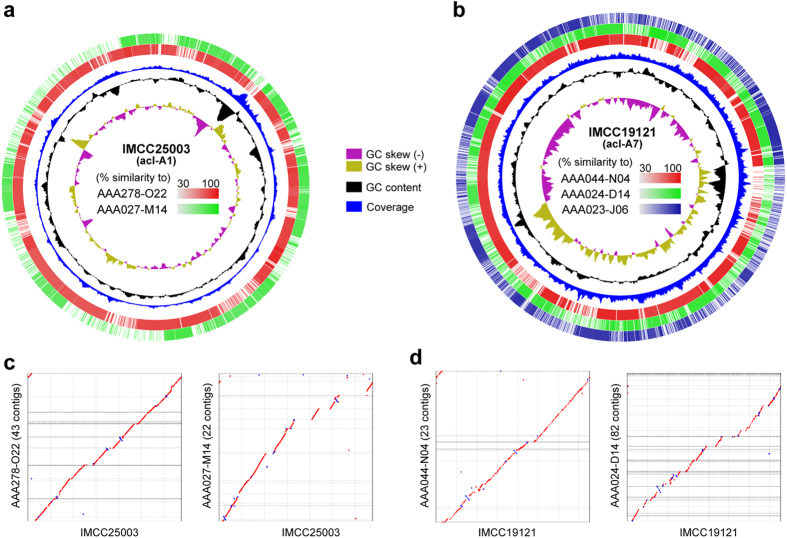
Comparison of the complete acI genomes and the SAGs of the same tribes. (**a** and **b**) BLASTn-based similarity analysis of the acI-A1 tribe (IMCC25003 and two SAGs) (**a**) and the acI-A7 tribe (IMCC19121 and three SAGs) (**b**). Similarity plots were drawn by BRIG with a similarity cutoff of 30%. Plots of GC skew and GC content were adopted from the respective genome maps (Supplementary Fig. S2). Coverage data are the same as those presented in Supplementary Fig. S3. (**c** and **d**) Synteny plots between the complete genomes and the SAGs of the tribes A1 (**c**) and A7 (**d**). Plots were drawn using MUMmer (Promer), after contig reordering by progressiveMauve.

Comparison of the acI genomes obtained in the present study and publicly available acI SAGs were performed. This comparison was possible for only two tribes, A1 and A7, because there were no SAGs of A4 and C1 tribes (Fig. 1b). The results showed that the complete acI genomes were highly similar to the SAGs of the same tribes (Fig. 3a and b), as could be expected from very high similarities (>99.5%) of 16 S rRNA genes (Fig. 1a). Further, contigs of the SAGs showed synteny to the complete acI genomes (Fig. 3c and d), suggesting a possibility that gene content and order were conserved well within the same tribes of the acI clade. However, it was also revealed that the two complete acI genomes harbored genomic regions that were underrepresented by SAGs (Fig. 3a and b). Although it remains unclear whether these regions are accessary genomes unique to the acI strains analyzed in the present study, or represent genomic regions inherently resistant to MDA, the comparison showed that the complete acI genomes expand the pan-genome sizes of the acI lineage.

### Genome streamlining

**Figure 4:**
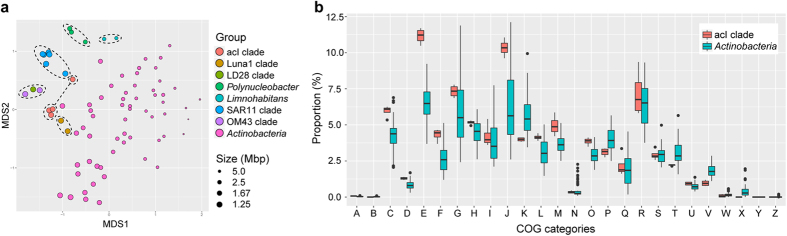
Comparative analysis of COG category distribution. (**a**) NMDS plot generated from COG category distribution of the acI clade, several representative freshwater bacterial groups, marine SAR11 and OM43 clades, and other actinobacterial strains (2D stress: 0.12). The diameter of symbols is inversely proportional to genome size. Strains of the same groups are enclosed by dashed ellipses. Note that the acI clade is indicated by two ellipses connected by a dashed line (only strain IMCC26077 in a smaller ellipse) and the LD28 and OM43 clades are enclosed together. (**b**) The distribution of the relative proportion of each COG category among the acI strains (*n* = 4) and other actinobacterial strains (*n* = 59). The names of COG categories are indicated by capital letters (in the x-axis) that can be found in Supplementary Fig. S2.

The acI genomes showed several features suggesting genome streamlining^30^, also suggested by SAG and MAG studies^19^,^20^,^22^,^23^. First, the lengths of the four complete genomes were short, ranging from 1.35 to 1.55 Mbp, and the genomic GC contents were low (45.5–51.3%) (Table 1). Second, the percentage of non-coding (intergenic) DNA was <5% for all acI genomes (Table 1), which fell within the lowest range among bacterial genomes^30^ and was comparable to those of marine SAR11 and OM43 strains, bacterial groups known for the smallest streamlined genomes^31^,^32^,^33^. In accordance with the high coding density, median lengths of intergenic regions were short, ranging from 9 (A7) to 14 (C1) bps. These lengths were comparable to those calculated for the acI genomes obtained by single-cell genomics or metagenome assembly^19^, and were in the lowest range among bacterial genomes^32^,^34^. Third, COG distribution of the acI genomes was distinct from the majority of actinobacterial genomes and was similar to that of streamlined genomes, including the genomes of the LD28 and Luna1 clades of freshwater bacteria^35^,^36^,^37^ and the SAR11 and OM43 clades of marine bacteria^31^,^33^, when analyzed using non-metric multidimensional scaling (NMDS) (Fig. 4a). COG categories K (transcription) and T (signal transduction) were underrepresented in the acI genomes (Fig. 4b), which suggest genome streamlining^38^. The fraction of genes assigned to the COG category P (inorganic ion transport and metabolism) was also low in the acI genomes. This category possibly includes traits related to r-strategists, and is therefore underrepresented during the evolution toward streamlined genomes^39^.

### Metabolic features of the acI strains deduced from the complete genomes

Metabolic reconstruction of the four acI genomes showed that the acI strains are aerobic chemoheterotrophs that had many metabolic pathways in common, but there were also many differences that might be related to niche diversification within the acI lineage.

### Carbon and energy

All strains had the Embden-Meyerhof-Parnas (EMP) glycolysis pathway (Table 2), which is in contrast to the Luna1 clade, another freshwater actinobacterial group with streamlined genomes. Genome analyses of two Luna1 clade strains, *Candidatus* Aquiluna sp. IMCC13023 and *Rhodoluna lacicola* MWH-Ta8^T^, showed that both strains lacked 6-phosphofructokinase, a key enzyme of the EMP pathway^35^,^36^. Although the E2 component of the pyruvate dehydrogenase complex was not annotated in the all acI genomes, it is possible that the E2 component of 2-oxoglutarate dehydrogenase complex may function as the E2 component for both dehydrogenase complexes as found for *Corynebacterium glutamicum*^40^. Interestingly, C1 had class I fructose-bisphosphate aldolase, whereas the acI-A strains had class II enzymes. The two classes utilize different reaction mechanisms and do not show sequence similarities^41^. Other than the EMP pathway, C1 had the upper steps of the semi-phosphorylative Entner-Doudoroff pathway (PWY-2221; MetaCyc). Although gluconate dehydratase, an enzyme involved in the pathway, was not annotated in the C1 genome, at least one of six putative mannonate dehydratases may function as gluconate dehydratase^42^. The oxidative branch of the pentose phosphate pathway (PPP) was found only in the A1 genome, whereas the non-oxidative branch was found in all genomes. Fructose-1,6-bisphosphatase, a key enzyme of gluconeogenesis, was found in A7 and C1, but not in A1 and A4 (Table 2).

Similarly to the two Luna1 clade strains, a complete TCA cycle was predicted in all acI strains (Table 2). But the glyoxylate shunt was not found in any of the genomes. Noticeably, succinate dehydrogenases of the strains had only two hydrophilic subunits and lacked two hydrophobic subunits (SdhC and SdhD), suggesting an inability to participate in the oxidative phosphorylation pathway as in complex II. Except for complex II, the complete oxidative phosphorylation pathway was predicted in the all genomes. The A4 and A7 genomes had a cytochrome *bd* complex in addition to cytochrome c oxidase. It is notable that proteins annotated as E1 components of the 2-oxoglutarate dehydrogenase complex had a partial E2 domain at their N-terminal. This domain architecture was also reported from the orthologous protein of *Mycobacterium tuberculosis*, which performed several chemical reactions regulated by acetyl-CoA^43^.

Differences among the acI strains were more marked in accessory carbon metabolism (Table 2). Formate dehydrogenase and a biosynthetic pathway for molybdenum cofactor (a putative cofactor of formate dehydrogenase) was found only in A7. Hexulose-6-phosphate synthase (HPS), one of the two key enzymes of the ribulose monophosphate pathway^44^, was predicted in A4 and C1. However, 6-phospho-3-hexuloisomerase, another key enzyme of the pathway, was not annotated. HPS found in the acI genomes may be involved in formaldehyde fixation and detoxification rather than methylotrophy, because methanol or methylamine dehydrogenase, representative enzymes for methylotrophy of freshwater methylotrophs such as the LD28 clade^37^, was not found in any of the acI genomes^44^. In congruence with the results obtained from SAGs^23^, synthesis and breakdown of polysaccharides were expected to be more active in A1 and A7 because the relevant enzymes such as alpha- and beta-galactosidase/glucosidase, glycogen synthase, and 1,4-alpha-glucan branching enzyme, were found only in IMCC25003 and IMCC19121. Enzymes involved in glucuronate interconversion, such as glucuronate isomerase, fructuronate reductase, altronate dehydrogenase, were predicted only in C1. These results implicated that the tribes of the acI clade may be niche-specialized in terms of carbon metabolism, in addition to the acquisition of carbon substrates suggested by SAG studies^23^.

Rhodopsin genes were found in all acI genomes and were all predicted to encode green-light absorbing rhodopsins with leucine residues at a key position^45^. But, the biosynthesis of retinal, an essential chromophore of rhodopsin, was complete in only A1 and A4 (Table 2). The A7 and C1 genomes had *crtEBIY* genes but lacked the *blh* gene encoding beta-carotene 15,15′-dioxygenase, the enzyme responsible for the final step of retinal biosynthesis. The lack of *blh* genes in the presence of rhodopsin was widespread among the acI lineage^23^ and was experimentally found to impair the proton pumping activity of rhodopsin in an actinobacterial strain of the Luna1 clade^46^.

### Biosynthesis of amino acids, nucleotides, and vitamin B compounds

All acI strains were predicted to synthesize 20 proteinogenic amino acids. Biosynthetic pathways for amino acids were shared among the strains, except for cysteine. A7 synthesized cysteine via *O*-acetylserine using serine *O*-acetyltransferase and *cysK*-encoded cysteine synthase A. The other three strains lacked serine *O*-acetyltransferase, but had genes for cysteine synthase B (*cysM*), a small sulfide carrier protein (*cysO*), and CysO-cysteine hydrolase (*mec*) in an operon structure, which suggested the presence of a novel cysteine biosynthetic pathway known from *M. tuberculosis*^47^. All acI strains lacked the assimilatory sulfate reduction pathway (Table 2), which indicates the growth requirement for reduced sulfur compounds, as reported from previous SAGs studies^22^,^23^.

Regarding nucleotide biosynthesis, C1 was distinct because the strain lacked several salvage pathway enzymes found in three other strains, including purine-nucleoside phosphorylase, adenosine deaminase, thymidine phosphorylase, thymidine kinase, and cytidine deaminase. *De novo* nucleotide synthesis was complete in all acI strains and was performed using common pathways.

The acI strains were auxotrophic for several vitamin B compounds, with some differences among the strains (Table 2). Vitamin B auxotrophy was also reported from a acI-B2 MAG^18^. All the four acI strains were auxotrophic for thiamine (vitamin B1), missing nearly all enzymes responsible for *de novo* and salvage pathways of thiamine biosynthesis. Furthermore, no transporters for thiamine were annotated explicitly. But, genes putatively annotated as coding for PnuC, nicotinamide mononucleotide transporter, were predicted in all acI genomes, and a THI element (thiamine pyrophosphate riboswitch) was found upstream of the genes. It seems highly probable that the products of these putative *pnuC* genes may function as PnuT, a Pnu-type thiamine transporter, when considering the sequence similarities between PnuC and PnuT and the existence of the THI element upstream of the genes^48^,^49^. The acI strains were also auxotrophic for biotin (vitamin B7). However, the biotin transport system substrate-specific component (BioY) protein was predicted in all genomes, without neighboring energy-coupling factor (ECF) proteins. These BioY proteins may function as a solitary biotin transporter^50^. The biosynthetic pathway for cobalamin (vitamin B12) was also not found in any of the acI strains. Instead, an ECF module preceded by cobalamin and AdoCbl riboswitches was predicted in each of the acI genomes, suggesting the role of these ECF modules as putative cobalamin transporters. In the C1 genome, an ECF module for cobalt/nickel transport was also predicted. Regarding riboflavin (vitamin B2) biosynthesis, strains A1, A7, and C1 had a complete gene set for riboflavin biosynthesis in an operon, and an FMN riboswitch was found immediately upstream of the operons. But, strain A4 lacked the operon and was predicted to be auxotrophic for riboflavin. Instead, the A4 genome had one more transporter gene putatively annotated as *pnuC* that showed only 20–25% sequence similarities to *pnuC* genes discussed above in relation to thiamine transport. Interestingly, the only FNM riboswitch of the A4 genome was predicted upstream of this gene, suggesting that the product of this putative *pnuC* gene may function as PnuX, a riboflavin transporter^51^. All acI strains had a gene for a dual-function enzyme riboflavin kinase/FMN adenylyltransferase responsible for the synthesis of FMN and FAD from riboflavin. Nicotinamide adenine dinucleotide (NAD; vitamin B3) was another cofactor that showed different biosynthetic capabilities among the acI strains. While other strains have both *de novo* and salvage pathways of NAD biosynthesis, A7 lacked the *de novo* pathway and possessed only a salvage pathway. All acI strains were prototrophic for other vitamin B compounds, such as pantothenic acid, pyridoxine, and folic acid.

### ABC transporters

All acI strains had ABC transporters for spermidine/putrescine, phosphate, and branched-chain amino acids in common (Table 2). However, no carbohydrate ABC transporters were shared among the all acI strains. Transporters for xylose were found in all acI-A strains, but not in C1. Alpha-glucoside transporters were predicted only in A1 and A7, in which alpha- and beta-glucosidase were found. These results are consistent with a previous SAG study that suggested the acI clade members have a capability to utilize N-rich organic compounds in common but are differentiated with regard to carbohydrate acquisition ability^23^.

## Discussion

The acI lineage, one of major constituents of freshwater bacterioplankton, has resisted cultivation so successfully that only two enrichment cultures have been reported, despite the ubiquity and abundance of the lineage^16^,^17^. In the present study, we obtained four novel cultures from four tribes (A1, A4, A7, and C1) of the acI lineage through HTC. Although the purity of the cultures were not confirmed thoroughly and the stable cultivation of the strains was not successful, these results showed the possibility of culturing the acI lineage members under laboratory conditions, and increased the number and diversity of cultured acI strains, which now includes strain IMCC26077, the first acI-C1 member. In addition, these acI cultures provided an opportunity to determine more complete acI genome sequences by increasing the number of cells available for WGA.

We applied WGA by MDA to more than hundreds of cells cultured by HTC and obtained the complete genomes of four acI members successfully. Single-cell genomics employing WGA by MDA have been affected by inescapable amplification bias that sometimes resulted in highly fragmented recovery of only part of a genome. Recently, some methods have overcome the amplification bias. Partition of single-cell DNA into numerous droplets in oil with an MDA reaction buffer decreased amplification bias significantly^52^. This approach seems to be applicable without prior cultivation of target cells, but has not been tested with prokaryote cells that have much smaller genome sizes than the eukaryotic cells used for the original study. Furthermore, a specifically fabricated microfluidic device is needed, which may be a problem for many microbiology labs. In another study, encapsulation and cultivation of microbial cells within agarose gel microdroplets followed by MDA was effective in reducing amplification bias, leading to nearly finished genomes of bacteria^27^. This method is high-throughput but requires expensive equipment. Further, the use of agarose gel to spatially contain the growth of target cells may be a barrier to the application of this method for oligotrophic environments because many bacterial lineages dominant in oligotrophic habitats do not grow on semisolid media. For example, the acI lineage of freshwater and the SAR11 clade of marine water columns have been cultured only in liquid media, but never been reported to form colonies on agar media^53^. When compared to the above methods, HTC followed by MDA, as developed in this study, would be more useful and widely applicable for the retrieval of complete genomes of diverse groups of bacteria inhabiting various environments for the following reasons: First, HTC has been very successful in isolating and cultivating diverse bacterial lineages from aquatic habitats^54^,^55^,^56^,^57^. Second, we have shown that the use of a large number of cells in MDA could reduce amplification bias, where minimum coverages are approximately 0.1 × average coverages (Table 1). Considering that raw data of bacterial genome sequencing can be obtained with >100–200 × coverage, our results suggest that at least ~10 × coverage would be attainable across the whole genome length, which could result in the recovery of nearly complete genome sequences. Third, contamination control, which is crucial for successful MDA of single-cell DNA but is difficult to perform^58^,^59^, would be easier when using a large number of cells cultured by HTC. We used a pre-decontaminated commercial MDA kit together with aseptic techniques that are routine in most microbiology labs for the MDA of HTC cultures, and there was little contamination in the MDA products.

The complete genomes obtained in this study, one acI-C and three acI-A genomes, enabled a comprehensive comparative analysis on genomic and metabolic features of the acI lineage, including the first acI-C1 tribe member cultured. The complete genomes facilitated reliable inter-genome comparisons and an unambiguous reconstruction of metabolic pathways in the acI members, without the uncertainties accompanying SAG- or MAG-based studies. For example, when analyzing SAGs with various genome recovery ratios, it is difficult to estimate genome sizes reliably and we cannot know whether the absence of a specific metabolic step reflects the actual missing step or the incomplete genome recovery.

The complete acI genomes, irrespective of the tribes, exhibited features characteristic of streamlined genomes: small genome lengths, high coding density with short intergenic regions, and COG category distribution biased against transcriptional regulation and signal transduction (Table 1 and Fig. 4)^30^,^38^. Loss of biosynthetic pathways of vitamin B compounds (B1, B7, and B12; Table 2) may also be an indication of genome streamlining that can confer selective advantages in natural habitats, but make it difficult to cultivate the acI lineage under laboratory conditions^18^,^60^,^61^. Genome streamlining has been found among many free-living bacterial groups of the ocean such as the SAR11 and SAR86 clades^32^,^62^ and the genus *Prochlorococcus*^63^ and is possibly an evolutionary strategy that may be successful under nutrient-poor conditions with low frequencies of environmental changes^30^. In this regard, the streamlined genomes of the acI members may underlie the ecological success of the lineage in freshwater habitats. When compared to some habitats where other groups of *Actinobacteria* are successful, such as soil and sediment, freshwater environments are characterized by relatively lower nutrient concentrations and the attenuation of physicochemical perturbations by unconstrained water mixing, where organisms with streamlined genomes may be preferred.

Based on a free-living lifestyle of the acI lineage^7^,^64^, the uptake and metabolic utilization of dissolved organic matter and inorganic nutrients is crucial for the survival of the lineage. Regarding these metabolic functions, the acI genomes had many features in common but also showed some differences among themselves. The acI strains had central carbon metabolic pathways in common, including the EMP pathway, TCA cycle, and non-oxidative PPP pathway. The strains also shared features related to inorganic nutrient utilization and the uptake of nitrogen-rich organic compounds, such as the lack of an assimilatory sulfate reduction pathway and the possession of transporters for phosphate, ammonium, spermidine/putrescine, and branched-chain amino acids (Table 2). In contrast, there were differences in the uptake capability for carbohydrates (Table 2). For example, transporters for glucose/mannose were predicted in A4 and C1, and the fructose transporter was annotated only in A4. Notably, this genome-based prediction corresponded partially with the uptake patterns of radiolabeled tracers. The proportions of acI cells that showed active incorporation was highest for leucine (a branched-chain amino acid), slightly over 50% for glucose, and very low for fructose^15^. In addition to differences in the uptake capabilities for carbohydrates, the acI strains also diverged in terms of accessory carbon metabolism, including alternative glycolytic pathways and polysaccharide utilization. It seems plausible that these differences among the tribes may be related to niche diversification within the acI lineage.

## Methods

### Sample collection, cultivation, and phylogenetic analysis

Water samples were collected from Lake Soyang three times (for details, refer to Table 1), transported immediately to laboratory at <10 °C, and used for HTC. To prepare media for HTC, water samples were filtered using a 0.2-μm polyethersulfone filter, autoclaved (1.5 h), aerated (2–4 h), and amended with a minute amount of nutrients (for details, refer to Supplementary Table S2)^57^. Based on the total prokaryotic numbers determined by counting DAPI (4′,6-diamidino-2-phenylinole)-stained cells using epifluorescence microscopy, water samples were diluted to 5 cells per ml in HTC media and dispensed into 48-well tissue culture plates (1 ml per well). After incubation at 15 °C for 4 weeks, 200 μl cultures from each well of the plates was stained with SYBR Green I (5 × final concentration) and examined by flow cytometry for cell counting (Guava easyCyte Plus, Millipore). Cultures of growth-positive (≥10^4^ cells ml^−1^) wells were used for phylogenetic analyses and stored at −80 °C after mixing with sterilized glycerol (10%, v/v).

Phylogenetic analyses of growth-positive cultures were based on PCR amplification and sequencing of 16 S rRNA gene sequences. Approximately 2 μl of culture were used as PCR templates after three cycles of freezing (−80 °C) and thawing (100 °C). PCR was performed using 27 F and 1492 R primers. Sanger sequencing of PCR products was performed using an 800 R primer. Determined sequences were inspected and trimmed manually using BioEdit and FinchTV, and aligned using an SINA online aligner^65^. Aligned sequences were imported into the SILVA database (SSURef NR 99, release 119) and inserted into the guide tree using the ARB program^66^. After curation, 16 S rRNA gene sequences of the acI strains and other reference strains/clones were exported using the “pos_var_ssuref:bacteria” filter and used to construct a maximum-likelihood tree using RAxML 8.1.17 with the GTRGAMMA model^67^. After tree building, a phylogenetic assignment of the sequences was performed following the scheme proposed by Newton *et al*.^4^.

The HTC cultures identified as belonging to the acI lineage were tested for sustained growth in laboratory conditions. Respective glycerol stocks (100–400 μl) were inoculated into 10–20 ml of the same media as used for the original HTC, and incubated at 15 °C in the dark. During incubation, bacterial growth was monitored using flow cytometry, and purity and identity of cultures were determined by RFLP and sequencing of amplified 16 S rRNA genes and microscopic examination. When the growth of the original strain was demonstrated, 1–2 rounds of transfers were performed in the same incubation conditions with an inoculum ratio of 1:10–1:20.

### Whole genome amplification, genome sequencing, and assembly

Glycerol stocks or cultures revived from glycerol stocks were used for WGA by MDA using a REPLI-g Single Cell Kit (Qiagen), according to manufacturer’s instructions. In brief, 4 μl of samples were lysed and used for the genome amplification reaction for 8 h at 30 °C in a total volume of 50 μl. The quantity and quality of MDA products were examined using a PicoGreen assay and gel electrophoresis. PCR amplification and sequencing of 16 S rRNA genes were performed using 50 nl of MDA products as templates to determine whether target genomes were amplified successfully without significant contamination.

Paired-end libraries for genome sequencing were generated from MDA products using Nextera library preparation methods. Genome sequencing was performed on the Illumina MiSeq platform (2 × 300 bp) at Chunlab, Inc. (South Korea). Assembly of Illumina sequencing data was performed using SPAdes 3.5.0 in a multi-cell mode, with read error and mismatch correction^68^. Single long contigs obtained from the assembly were circularized manually by PCR and sequencing that targeted the contig end regions. To avoid an erroneous gap-filling, PCR primers were designed to be unique not only in the single long contigs but also in the all contigs assembled by SPAdes. In addition, the genomic regions determined by sequencing of PCR products were analyzed by BLASTn, which confirmed there were no SPAdes-assembled contigs having a region (≥50 bp) identical to the PCR-closed sequences.

Coverage variation across the whole genomes were inspected by mapping the raw sequencing reads onto the complete genome sequences^26^. Raw reads were trimmed using Trimmomatic ver. 0.35 (ref. 69) (with options as follows; ILLUMINACLIP:NexteraPE-PE.fa:2:30:10:8:keepBothReads LEADING:5 TRAILING:5 SLIDINGWINDOW:4:15 MINLEN:100). The surviving paired reads were mapped using BWA-MEM^70^. Conversion into BAM format and the removal of putative duplicates were performed using Picard Tools. After indexing by samtools, IGV was used for manual inspection of mapping results and coverage visualization with a 25-bp window. Coverage per base resolution was obtained by samtools with a depth option.

Genome-scale analysis of sequence similarities among the acI genomes was performed using the Artemis Comparison Tool. Comparison files were created by BLASTn, and only the matches satisfying length (≥60 bp) and score (≥50) cutoff were visualized. Comparison between the complete acI genomes and SAGs of the same tribes were performed using Blast Ring Image Generator (BRIG)^71^. BLASTn results were displayed with a similarity cutoff of 30%. Synteny plots between the complete acI genomes and SAGs were drawn using the Promer program of the MUMmer^72^, after contig reordering by progressiveMauve^73^.

### Annotation and comparative analysis of genomes

Annotation of the genome sequences was performed using the IMG-ER pipeline, with the Isolate Genome Gene Calling method. Metabolic pathways encoded in the genomes were examined using KEGG and MetaCyc pathways as provided by the IMG system. If necessary, protein sequences were analyzed using BLASTp against the NCBI nr database. Riboswitches were searched using the cmsearch program of Infernal based on covariance models downloaded from Rfam^74^. The models used were RF00059.cm (THI element), RF00059.cm (FMN riboswitch), RF00174.cm (Cobalamin riboswitch), and RF01482.cm (AdoCbl riboswitch).

For comparative analysis of COG category distribution, 79 strains were selected: 4 acI strains from the present study, 2 actinobacterial Luna1 clade strains, 1 LD28 clade (*Candidatus* Methylopumilus planktonicus) strain, 3 *Polynucleobacter* strains, 2 *Limnohabitans* strains, 6 SAR11 clade strains, 2 OM43 clade strains, and 59 other actinobacterial strains (Supplementary Table S3). The actinobacterial strains were selected with a bias toward smaller genomes, but retaining the phylogenetic diversity of the phylum. Each actinobacterial strain was selected from the families of the phylum *Actinobacteria*, based on a phylogenetic scheme available at http://www.ezbiocloud.net/ezgenome. The smallest genomes within each family were selected, excluding genomes with too many scaffolds or pseudogenes. When the smallest genome of a family was shorter than 2 Mbp, each strain was further selected from the all genera of the family. The genus *Tropheryma* was excluded because this genus represents obligately parasitic strains. The COG category distribution data of selected strains were downloaded from IMG, using the ‘Abundance profiles’ function. The number of CDSs assigned to each COG category was normalized by the number of total CDSs of each genome, and then square-root transformed. NMDS analysis was performed using the R package ‘*vegan*’. Non-transformed data of the acI strains and other actinobacterial strains were used for comparison based on boxplot.

Protein sequences predicted in the genomes were downloaded from IMG and used for construction of a phylogenetic tree by PhyloPhlAn^75^. The protein sequences were also used for comparative pan- and core-genome analyses using GET_HOMOLOGUES software with the orthoMCL algorithm^76^. All parameters were default, except for minimum coverage in BLAST pairwise alignments (70%). After clustering, the proportion of shared PCs was calculated by dividing the number of shared PCs by the average number of total PCs of the genomes compared. AAI among the genomes were also obtained from GET_HOMOLOGUES. Clustering of the genomes based on AAI was performed using the UPGMA method with the ‘hclust’ function of R.

## Additional Information

**Accession codes:** The whole genome sequences reported in this study are available in the Joint Genome Institute’s Integrated Microbial Genome database (http://img.jgi.doe.gov/cgi-bin/w/main.cgi), under the accession numbers 2602042019 (IMCC25003), 2602042020 (IMCC26103), 2606217181 (IMCC19121), and 2602042021 (IMCC26077). The sequences were also deposited in GenBank under the accession numbers CP015603 (IMCC25003), CP015604 (IMCC26103), CP015605 (IMCC19121), and CP015606 (IMCC26077).

**How to cite this article:** Kang, I. *et al*. The first complete genome sequences of the acI lineage, the most abundant freshwater *Actinobacteria*, obtained by whole-genome-amplification of dilution-to-extinction cultures. *Sci. Rep.* **7**, 42252; doi: 10.1038/srep42252 (2017).

**Publisher's note:** Springer Nature remains neutral with regard to jurisdictional claims in published maps and institutional affiliations.

## Supplementary Material

Supplementary Information

## Figures and Tables

**Table 1:** ECG and EGCG derivatives and their autophagic activation.

Strain (Tribe)		IMCC25003 (acI-A1)	IMCC26103 (acI-A4)	IMCC19121 (acI-A7)	IMCC26077 (acI-C1)
Isolation	Date isolated	Jun, 2013	Apr, 2014	Oct, 2011	Apr, 2014
	Latitude (N)	37° 55´ 52.5˝	37° 56´ 50.6˝	37° 56´ 37.5˝	37° 56´ 50.6˝
	Longitude (E)	127° 53´ 10.7˝	127° 49´ 7.9˝	127° 50´ 35.6˝	127° 49´ 7.9˝
	Depth (m)	5	50	1	1
	Temperature (°C)	24.1	6.1	17.2	12.3
Genome features	Size (Mbp)	1.354	1.457	1.506	1.552
	%GC	49.1	47.0	45.5	51.3
	Coding density (%)	96.0	95.8	96.0	95.5
	rRNA	3	3	3	3
	tRNA	40	38	38	48
	CDS	1,358	1,434	1,487	1,517
	Coverage*	608 (76–4,145)	331 (30–2,437)	682 (92–2,740)	376 (33–3,003)

Average coverage. Minimum and maximum coverages (per base resolution) are indicated within parentheses

**Table 2:** Metabolic features of the acI strains deduced from their genome sequences.

Strain (Tribe)		IMCC25003 (acI-A1)	IMCC26103 (acI-A4)	IMCC19121 (acI-A7)	IMCC26077 (acI-C1)
Photoheterotrophy	Rhodopsin	**•**	**•**	**•**	**•**
	Retinal biosynthesis	**•**	**•**	**×**	**×**
Carbon metabolism	EMP	**•**	**•**	**•**	**•**
	ED (semi-phosphorylative)	**×**	**×**	**×**	**•**
	PPP, oxidative	**•**	**×**	**×**	**×**
	PPP, non-oxidative	**•**	**•**	**•**	**•**
	TCA	**•**	**•**	**•**	**•**
	Gluconeogenesis	**×**	**×**	**•**	**•**
	Formate dehydrogenase	**×**	**×**	**•**	**×**
	α, β-Glucosidase/galactosidase	**×**	**•**	**×**	**•**
	Glycogen synthesis	**•**	**×**	**•**	**×**
Vitamin and cofactor	Thiamine	**×**	**×**	**×**	**×**
	Biotin	**×**	**×**	**×**	**×**
	Cobalamin	**×**	**×**	**×**	**×**
	Riboflavin	**•**	**×**	**•**	**•**
	Niacin	**•**	**•**	**×**	**•**
	Pantothenic acid	**•**	**•**	**•**	**•**
	Pyridoxine	**•**	**•**	**•**	**•**
	Folic acid	**•**	**•**	**•**	**•**
	Molybdenum cofactor	**×**	**×**	**•**	**×**
ABC transporters	Spermidine/Putrescine	**•**	**•**	**•**	**•**
	Glycine betaine/Proline	**×**	**×**	**•**	**×**
	Osmoprotectant	**•**	**×**	**×**	**×**
	Sorbitol/Mannitol	**•**	**×**	**×**	**×**
	α-Glucoside	**•**	**×**	**•**	**×**
	Raffinose/Stachyose/Melibiose	**×**	**•**	**•**	**•**
	Galactose oligomer/Maltooligosaccharide	**×**	**×**	**•**	**×**
	Glucose/Mannose	**×**	**•**	**×**	**•**
	Fructose	**×**	**•**	**×**	**×**
	Ribose/D-Xylose	**•**	**×**	**×**	**•**
	D-Xylose	**•**	**•**	**•**	**×**
	Branched-chain amino acid	**•**	**•**	**•**	**•**
	Iron complex	**•**	**×**	**×**	**•**
	Cobalt	**×**	**×**	**×**	**•**
Inorganic nutrient utilization	Phosphate transporter	**•**	**•**	**•**	**•**
	Ammonium transporter	**•**	**•**	**•**	**•**
	Assimilatory sulfate reduction	**×**	**×**	**×**	**×**

EMP, the Embden-Meyerhof-Parnas pathway; ED, the Entner-Doudoroff pathway; PPP, pentose phosphate pathway; TCA, tricarboxylic acid cycle. ^•^ Presence; × Absence.
