# Erratum: The first complete genome sequences of the acI lineage, the most abundant freshwater *Actinobacteria*, obtained by whole-genome-amplification of dilution-to-extinction cultures

**DOI:** 10.1038/srep46830

**Published:** 2017-05-26

**Authors:** Ilnam Kang, Suhyun Kim, Md. Rashedul Islam, Jang-Cheon Cho

Scientific Reports 7: Article number: 42252; 10.1038/srep42252 published online: 02 10 2017; updated: 05 26 2017.

This Article contains errors. The title of Table 1 is incorrect, where:

‘Table 1: ECG and EGCG derivatives and their autophagic activation’.

should read:

“Table 1. Overall features of four acI strains and their genomes’.

Additionally, the legend of Table 1:

‘Average coverage. Minimum and maximum coverages (per base resolution) are indicated within parentheses’.

should read:

‘*Average coverage. Minimum and maximum coverages (per base resolution) are indicated within parentheses’.

