# Supplementary material for: The first complete genome sequences of the acI lineage, the most abundant freshwater Actinobacteria, obtained by whole-genome-amplification of dilution-to-extinction cultures
Source: Sci Rep. 2017 Feb 10;7:42252. doi: 10.1038/srep42252 (PMC5301498; doi:10.1038/srep42252)
Supplement: Supplementary Information [file srep42252-s1.pdf]

Supplementary Information

The first complete genome sequences of the acI lineage, the most abundant freshwater *Actinobacteria*, obtained by whole-genome-amplification of dilution-to-extinction cultures

Ilnam Kang, Suhyun Kim, Md. Rashedul Islam, and Jang-Cheon Cho*

Department of Biological Sciences, Inha University, Incheon 22212, Republic of Korea

Correspondence: Jang-Cheon Cho, Department of Biological Sciences, Inha University, Incheon 22212, Republic of Korea. E-mail: chojc@inha.ac.kr

Supplementary Figure S1. Revival and transfer cultures of the acI strains.

Supplementary Figure S2. Genome maps of the acI strains.

Supplementary Figure S3. Coverage variation across the complete acI genomes.

Supplementary Table S1. Lengths and coverages of the ten longest contigs obtained by SPAdes assembly

Supplementary Table S2. Chemicals added to the HTC media

Supplementary Table S3. List of strains used for the analysis of the COG category distribution

Supplementary References

Supplementary Figures

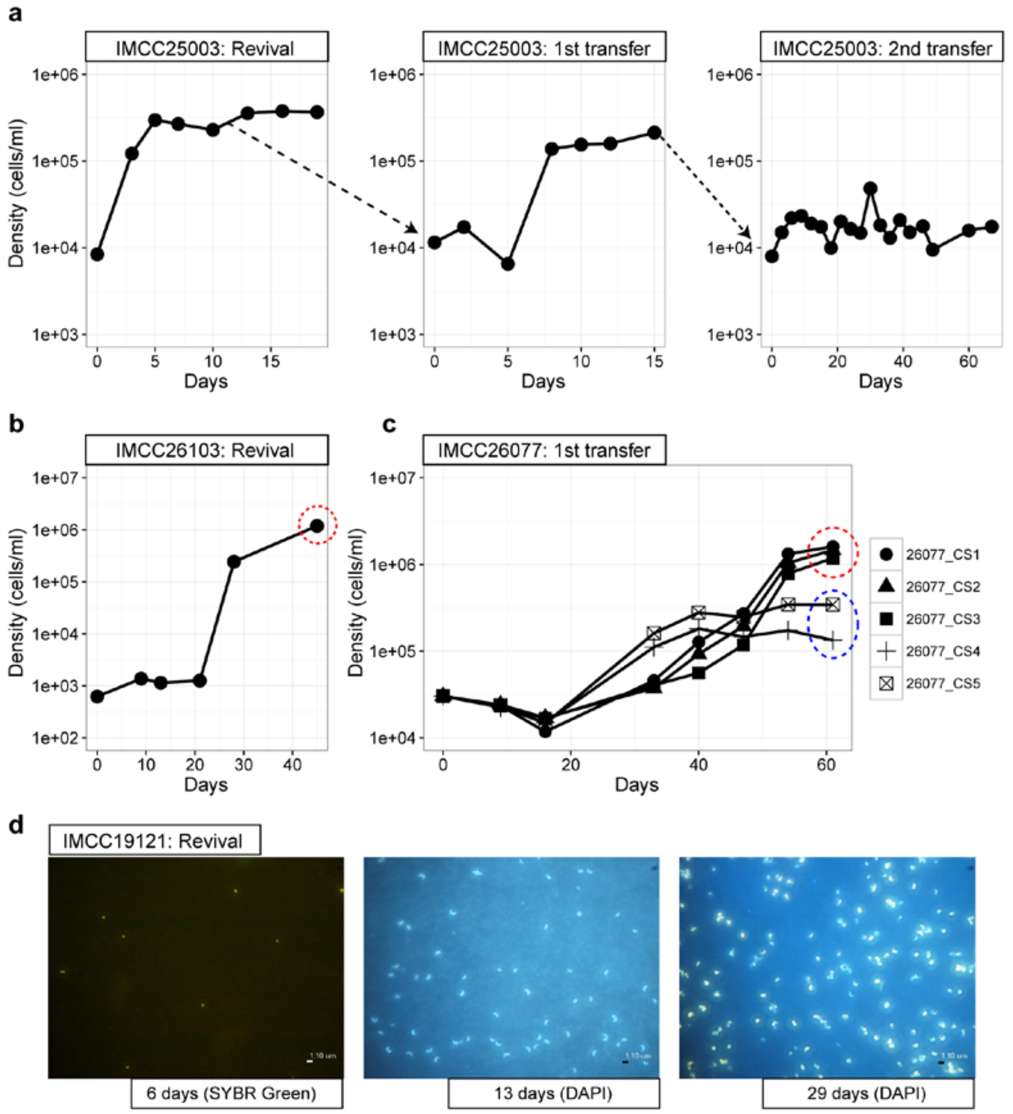

Supplementary Figure S1. Revival and transfer cultures of the acI strains. (**a**) Growth curves of IMCC25003. Dashed arrows indicate the transfer events. (**b**) Revival culture of IMCC26103. The culture surrounded by a red dashed circle was dominated by a bacterial strain other than IMCC26103. (**c**) Growth curves of IMCC26077 in media amended with different carbon sources. Cultures surrounded by red dashed circles were dominated by other bacterial strains. IMCC26077 dominated the cultures surrounded by blue dashed circles, but was outgrown by other bacterial strains in the next transfer. (**d**) Microscopic images of the IMCC19121 revival culture. IMCC19121 (small cocci) was dominant until 6 days after inoculation, and was gradually replaced by other bacterial strains (curved rod) during continued incubation. Scale bar (lower right), 1.1 µm.

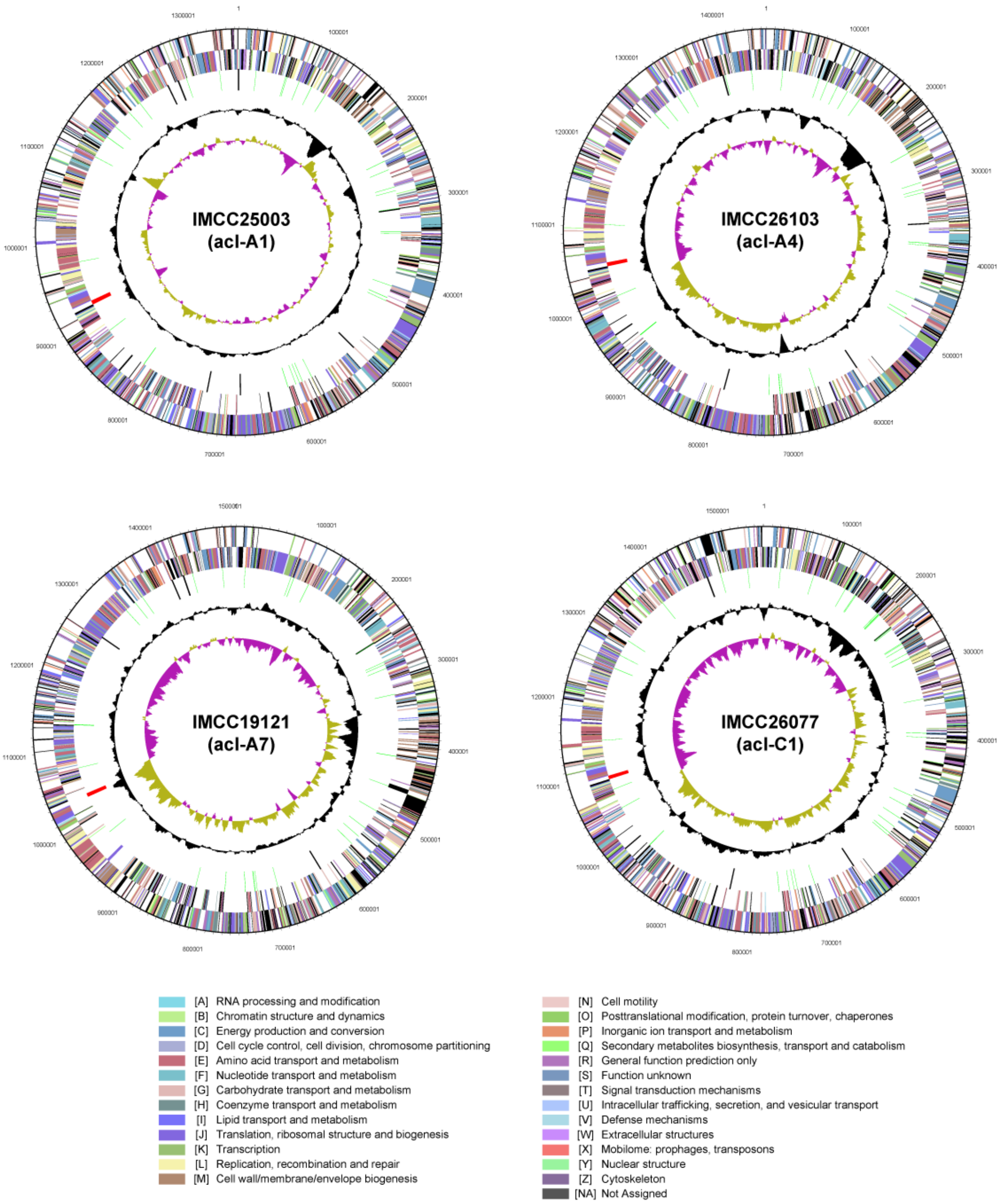

Supplementary Figure S2. Genome maps of the acI strains. From outside to the center: Genes on the forward strand (color by COG categories), genes on the reverse strand (color by COG categories), RNA genes (tRNAs green, rRNAs red, other RNAs black), GC content, and GC skew. Color designation of COG categories are shown at the bottom.

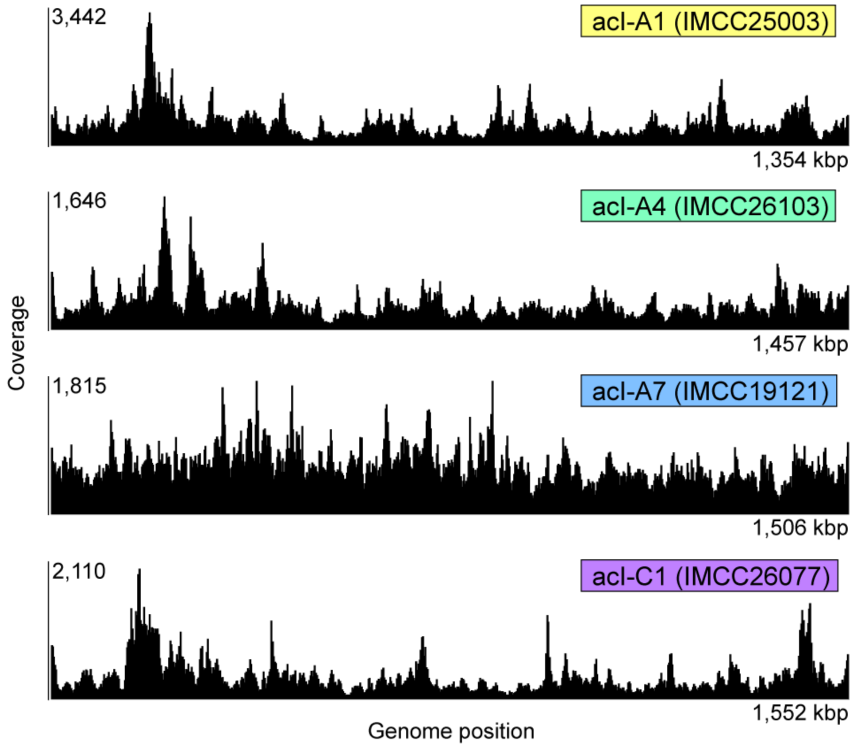

Supplementary Figure S3. Coverage variation across the complete acI genomes, calculated using a 25-bp window. Bar heights were normalized in each genome and do not correspond to actual values.

Supplementary Tables

Supplementary Table S1. Lengths and coverages of the ten longest contigs obtained by SPAdes assembly

IMCC25003		IMCC26103		IMCC19121		IMCC26077	
Length (bp)	Coverage	Length (bp)	Coverage	Length (bp)	Coverage	Length (bp)	Coverage
1,354,074	255.9	1,455,315	155.5	1,506,829	361.6	1,551,865	177.4
30,222	9.6	295,860	12.3	212,437	9.9	22,893	5.3
29,580	6.5	116,792	13.0	171,463	7.6	22,105	6.8
24,775	5.1	69,444	14.5	152,258	6.4	16,760	4.4
21,366	4.1	55,646	14.3	131,094	8.1	15,576	4.2
19,796	12.8	54,529	7.8	104,644	5.3	14,763	5.2
15,381	4.2	49,984	5.7	87,342	5.5	14,096	4.8
11,805	4.1	48,864	5.8	85,904	4.9	14,021	3.6
11,655	4.2	48,476	8.4	79,363	8.4	13,430	3.7
10,071	4.3	45,655	7.4	78,832	4.6	12,052	5.4

Supplementary Table S2. Chemicals added to the HTC media

Chemical	Final concentration	
	IMCC25003 & IMCC19121	IMCC26103 & IMCC26077
NH_4Cl	10 µM	10 µM
KH_2PO_4	10 µM	10 µM
Pyruvic acid	50 µM	50 µM
D-Glucose	5 µM	5 µM
N-Acetyl- D-glucosamine	5 µM	5 µM
Acetic acid	5 µM	-
$FeCl_3$	1 µM	-
D-Ribose	-	5 µM
Methyl alcohol	-	5 µM
Methionine	1 µM	-
Glycine	1 µM	-
Cysteine	1 µM	-
Amino acids mixture*	-	100 nM, each
Vitamin mixture†	1 ×	1 ×
Trace metal‡	-	1 ×

*Mixture of 20 proteinogenic amino acids
†See Stingl *et al.* (2008) for a detailed composition.
‡See Carini *et al.* (2013) for a detailed composition.

Supplementary Table S3. List of strains used for the analysis of the COG category distribution

taxon_oid	Genome Name	Group	Genome Size	Scaffold Count	GC (%)	Coding density (%)
2602042019	IMCC25003	acI	1353947	1	49	96.02
2602042020	IMCC26103	acI	1456516	1	47	95.80
2606217181	IMCC19121	acI	1506415	1	45	96.04
2602042021	IMCC26077	acI	1551612	1	51	95.53
2602042091	*Candidatus* Aquiluna sp. IMCC13023	Luna1	1361614	1	52	94.43
2504643007	*Rhodoluna lacicola* MWH-Ta8	Luna1	1430433	1	52	95.16
2663762675	*Candidatus* Methylopumilus planktonicus MMS-2-53	LD28	1356411	1	37	94.72
2634166443	*Polynucleobacter necessarius asymbioticus*	*Polynucleobacter*	2026292	1	45	93.56
2517572181	*Polynucleobacter* sp. MWH-UH21B	*Polynucleobacter*	2040808	1	45	94.08
2687453598	*Polynucleobacter wuianus* QLW-P1FAT50C-4	*Polynucleobacter*	2232205	1	45	93.29
2522572097	*Limnohabitans* sp. Rim28	*Limnohabitans*	3411710	73	59	92.41
2684622883	*Limnohabitans* sp. Rim47	*Limnohabitans*	2955075	1	59	92.53
2623620844	*Candidatus* Pelagibacter ubique SAR11 HTCC1002	SAR11	1327604	5	30	95.91
2623620681	*Candidatus* Pelagibacter ubique SAR11 HTCC1062	SAR11	1308759	1	30	96.50
2503283017	*Candidatus* Pelagibacter sp. HTCC7211	SAR11	1456888	1	29	96.70
2521172709	Alphaproteobacterium sp. HIMB5	SAR11	1343202	1	29	96.76
650716017	*Candidatus* Pelagibacter sp. IMCC9063	SAR11	1284727	1	32	94.93
2503283019	*Rickettsiales* sp. HIMB114	SAR11	1237371	1	30	96.98
2623620328	*Methylophilales* sp. HTCC2181	OM43	1304428	3	38	95.15
2503283018	Marine bacterium *Betaproteobacteria* HIMB624	OM43	1333209	1	35	96.43
644736322	*Acidimicrobium ferrooxidans* ICP, DSM 10331	*Actinobacteria*	2158157	1	68	91.53
639633001	*Acidothermus cellulolyticus* 11B	*Actinobacteria*	2443540	1	67	90.22
2524614758	*Actinobaculum urinale* DSM 15805	*Actinobacteria*	1920341	29	51	87.06
643886017	*Actinomyces coleocanis* DSM 15436	*Actinobacteria*	1719346	40	50	90.81
2516493031	*Actinopolyspora mortivallis* HS-1, DSM 44261	*Actinobacteria*	4233350	1	69	86.58
2510065062	*Actinospica robiniae* DSM 44927	*Actinobacteria*	9918887	1	70	87.59
2643221834	*Aeromicrobium* sp. Leaf245	*Actinobacteria*	3465317	16	72	92.02
2541047096	*Alloscardovia omnicolens* F0580	*Actinobacteria*	1789070	44	47	88.16
646564505	*Arcanobacterium haemolyticum* CCM, DSM 20595	*Actinobacteria*	1986154	1	53	90.51
2562617091	*Atopobium vaginae* DSM 15829	*Actinobacteria*	1430526	6	43	88.74
643692008	*Beutenbergia cavernae* HKI 0122, DSM 12333	*Actinobacteria*	4669183	1	73	92.97
2524023203	*Bifidobacterium magnum* DSM 20222	*Actinobacteria*	1825255	22	59	88.37
2600255077	*Blastococcus* sp. AP3	*Actinobacteria*	3864252	78	73	91.18
2548877016	*Brevibacterium massiliense* 5401308	*Actinobacteria*	2349262	26	62	90.96
2565956803	*Candidatus* Microthrix parvicella Bio17-1	*Actinobacteria*	4202850	13	66	90.76
644736339	*Catenulispora acidiphila* ID139908, DSM 44928	*Actinobacteria*	10467782	1	70	89.49
2585427833	*Cellulomonas* sp. HZM	*Actinobacteria*	3559280	77	73	92.07
2562617146	*Collinsella intestinalis* DSM 13280	*Actinobacteria*	1804297	55	62	89.60
2522572195	*Conexibacter woesei* Iso977N	*Actinobacteria*	5695417	9	72	94.05
2558860387	*Coriobacteriaceae* sp. BV3AC1	*Actinobacteria*	1801377	21	49	89.47
2515154055	*Corynebacterium caspium* DSM 44850	*Actinobacteria*	1843677	8	50	90.99
644736346	*Cryptobacterium curtum* 12-3, DSM 15641	*Actinobacteria*	1617804	1	51	88.83
2510065084	*Cryptosporangium arvum* YU 629-21, DSM 44712	*Actinobacteria*	9195993	1	72	91.62

taxon_oid	Genome Name	Group	Genome Size	Scaffold Count	GC (%)	Coding density (%)
2541047015	*Dermabacter* sp. HFH0086	*Actinobacteria*	2361288	8	63	91.43
2523533584	*Dermatophilus congolensis* DSM 44180	*Actinobacteria*	2623310	11	59	90.25
2547132114	*Dietzia alimentaria* 72	*Actinobacteria*	3352817	39	67	91.62
2600255000	*Gardnerella vaginalis* 00703Dmash	*Actinobacteria*	1490797	11	43	88.78
2524614582	*Gordonia shandongensis* DSM 45094	*Actinobacteria*	3330647	33	69	92.16
2516653079	*Haloglycomyces albus* DSM 45210	*Actinobacteria*	3535419	1	60	86.05
2545824624	*Ilumatobacter coccineum* YM16-304	*Actinobacteria*	4830181	1	67	93.12
2505679068	*Isoptericola variabilis* 225	*Actinobacteria*	3307740	1	74	92.73
2531839443	*Janibacter hoylei* PVAS-1	*Actinobacteria*	3139099	122	71	86.04
2509601042	*Jiangella gansuensis* YIM 002, DSM 44835	*Actinobacteria*	5585780	1	71	91.37
644736376	*Jonesia denitrificans* 55134, DSM 20603	*Actinobacteria*	2749646	1	58	91.83
640753031	*Kineococcus radiotolerans* SRS30216	*Actinobacteria*	4956672	3	74	91.06
644736380	*Kytococcus sedentarius* 541, DSM 20547	*Actinobacteria*	2785024	1	72	91.28
2547132085	*Mycobacterium bovis* BCG-Denmark TMC 1010, ATCC 35733	*Actinobacteria*	4255552	32	65	89.83
2524614512	*Nakamurella lactea* DSM 19367	*Actinobacteria*	5820860	27	69	91.63
2515154141	*Nonomuraea coxensis* DSM 45129	*Actinobacteria*	8996452	101	72	90.88
2561511089	*Paraoerskovia marina* DSM 21750	*Actinobacteria*	2948319	11	71	92.88
649989980	*Parascardovia denticolens* B3028, F0305	*Actinobacteria*	1891248	2	58	89.82
2524614558	*Patulibacter americanus* DSM 16676	*Actinobacteria*	4470560	32	74	91.11
2523533555	*Propionimicrobium lymphophilum* DSM 4903	*Actinobacteria*	2035623	26	56	91.26
2524614761	*Pseudoclavibacter soli* DSM 23366	*Actinobacteria*	2223047	9	64	93.00
645058800	*Rothia mucilaginosa* ATCC 25296	*Actinobacteria*	2255158	25	60	82.97
2524614511	*Ruania albidiflava* DSM 18029	*Actinobacteria*	4174923	60	70	91.13
637000248	*Rubrobacter xylanophilus* DSM 9941	*Actinobacteria*	3225748	1	70	93.63
2517093017	*Saccharomonospora halophila* 8	*Actinobacteria*	3685023	116	71	88.37
2517287023	*Salinispora pacifica* DSM 45548	*Actinobacteria*	5097790	2	70	89.13
647533207	*Scardovia inopinata* F0304	*Actinobacteria*	1804013	9	49	85.80
646564566	*Segniliparus rotundus* CDC 1076, DSM 44985	*Actinobacteria*	3157527	1	67	91.29
2558860160	*Solirubrobacterales* bacterium URHD0059	*Actinobacteria*	6572728	17	72	92.52
2516653049	*Sporichthya polymorpha* DSM 43042	*Actinobacteria*	5500153	1	71	92.38
2524614564	*Streptomyces* sp. TAA040	*Actinobacteria*	5311812	74	73	86.13
637000319	*Thermobifida fusca* YX	*Actinobacteria*	3642249	1	68	85.79
646564581	*Thermobispora bispora* R51, DSM 43833	*Actinobacteria*	4189976	1	72	84.55
646311963	*Thermomonospora curvata* DSM 43183	*Actinobacteria*	5639016	1	72	85.33
646564587	*Tsukamurella paurometabola* 33, DSM 20162	*Actinobacteria*	4479724	2	68	93.46
2524614768	*Yaniella halotolerans* DSM 15476	*Actinobacteria*	2775456	6	56	91.64

Supplementary References

Carini P, Steindler L, Beszteri S, Giovannoni SJ. (2013). Nutrient requirements for growth of the extreme oligotroph '*Candidatus* Pelagibacter ubique' HTCC1062 on a defined medium. *ISME J* **7**: 592-602.

Stingl U, Cho J-C, Foo W, Vergin K, Lanoil B, Giovannoni S. (2008). Dilution-to-extinction culturing of psychrotolerant planktonic bacteria from permanently ice-covered lakes in the McMurdo Dry Valleys, Antarctica. *Microb Ecol* **55**: 395-405.
